# The Deaf and the Dumb

**Published:** 1889-09-28

**Authors:** 


### The Deaf and the Dumb.

Once Mr. Haweis discussed the relative disadvantages of deafness and blindness. He assumed that most people would hold that the loss of sight was the greater misfortune, and was at some pains to prove that in reality the loss of hearing separated the sufferer from the rest of mankind more than the want of any other sense. He did not, however, touch on one very serious *sequela* of deafness, the dumbness that so frequently results. Formerly, the two misfortunes went almost inseparably together, and even now, when the old idea that the loss of hearing was accompanied by some actual injury to the vocal organs, it is difficult to prevent those who become deaf in childhood from forgetting the art of speech also, and developing into deaf mutes. Much depends on the degree of deafness, but still more on the age at which it develops. An adult has the habit of speaking too firmly implanted in him to lose it easily, and, moreover, he has a more earnest desire to maintain some degree of companionship with his fellows. Certain peculiarities of pronunciation do indeed appear in course of time, especially with words not in the commonest use; the vocabulary becomes limited by slow degrees, but for all practical purposes it is as large as need be.

With a child the case is different. A child does not realise the value either of the power of hearing it has lost or the power of speech it may lose. It does not strive to hear, and therefore may be looked upon as totally deaf when the defect of the ear is such as would make an older person be regarded as "hard of hearing." Thus, not trying to hear, it forgets how to talk. The words of its limited vocabulary are by no means firmly fixed in its memory; it soon forgets them, and it adds no more. It becomes a deaf mute, and very possibly is accredited with very limited intelligence on account of its isolation from the ideas of others. Dr. James Erskine, surgeon to the Glasgow Ear Institution, has come to the conclusion that if a child becomes deaf before the age of four years, dumbness almost always follows, and that speech is very often lost even if the deafness is acquired between the ages of four and seven. Children of that age who thus become dumb are not by any means totally deaf; they do not take the trouble to listen to what is said around them, and thus they lose the power of speech. Dr. Erskine lays it down as a rule that the younger the child the smaller is the degree of defective hearing that will produce dumbness.

Deafness is more commonly acquired than inherited. It may result from many diseases, and though such deafness may go off with renewed strength it is quite as likely to become permanent. A very common and irremediable cause of deafness is the frequently neglected ailment commonly called "running ears." This may go on for some time unnoticed, or, at least, disregarded by the uneducated, who do not realise that the running implies an inflammation which is gradually destroying the middle ear and the real apparatus of hearing. They would be much more disturbed by an injury to the comparatively useless external ear.

Deafmutism—that is, deafness associated with dumbness—is not a common disease, and perhaps receives too little attention on that account. The proportion of deaf mutes to the hearing population is about seven in ten thousand. The number is smallest in Holland—3·35 per 10,000; and highest in Switzerland—24·5 per 10,000. The reason of this great difference between countries not very far apart is to be found in their configuration. Not that hills produce deafness, but that in hilly countries the inhabitants, owing to the difficulty of travelling, are more given to marrying near relations. If consanguineous marriages do not absolutely produce disease in the offspring, as has often been said, they at least tend to give prominence to any hereditary taint in the constitution which might almost have disappeared under some counter acting influence derived from either parent. The connection between deafmutism and consanguinity is very clearly shown in the statistics of the subject. It is very common among Jews, with whom close intermarriages are of necessity common. Among Christian communities Catholics suffer less from it than any others, consanguineous marriages being forbidden by the Church, though the rule may occasionally be relaxed by special dispensation. In China, the law prohibits such marriages, and there the number of deaf mutes is remarkably small. In the United States the increase of deaf mutes in proportion to the general population is surprising; indeed, rather alarming. The total population has doubled during the last forty years; but in that time the deaf-mute population has quadrupled. The reason of this is to be found in the fact that, whereas in old times the deaf mute was one apart from others, he is now—thanks to the education which enables him to earn his living—able to take his place among the ranks of citizens. Among other rights thus won is that of marriage, and not unnaturally he chooses as his wife a deaf mute like himself. Their children are almost certain to inherit their parents' deficiency, and so a regular deaf mute population becomes established. It is not certain that this is altogether to be regretted. While admitting that deaf mutes may be, and are, praiseworthy members of society, it is not to be desired that their defect should spread to families yet untainted with it. It is better that they should marry among themselves, and so, to use a cruel sounding but sensible country phrase, "save spoiling a better pair." Moreover, even this equality of condition between husband and wife is likely to conduce to happiness. Certain stern sanitarians, too ready to sacrifice the individual to the race, might forbid marriage altogether to deaf mutes. For most people, however, it will be enough to provide that they shall have the opportunity of becoming useful members of society. The rest may be left to themselves and Providence.

Of the two methods of teaching the deaf—the oral and the digital—the first is incomparably the best. By it the dumb are absolutely taught to speak; the disused vocal organs are brought into play. Though deaf, they are no longer deaf mutes, and guess their words from the movements of articulation. This brings them into contact with the world at large, and no longer confines them to the companionship of those who have learned the finger signs. Spain was one of the first countries in which the oral method was systematically taught, and a visitor to a Spanish school observed how in deaf mutes gathered from various districts their articulate utterances bore the accent of their province, although they had never heard it spoken. The first school in the education of the deaf and dumb established in Britain was that in Edinburgh, which gave its name to the district of "Dumbiedykes." Thomas Braidwood, who founded it, taught the oral method, but the difficulty of teaching lip-reading, compared with showing the digital signs, has caused that method to be more or less neglected till recently. It is still difficult to obtain education for deaf-mute children on account of the expense of the special instruction necessary. The comparatively small number of deaf-mute children to be found in any town increases the difficulty of providing for them unless their parents are fairly well off. Still, the School Boards of London, Leeds, Sheffield, Dundee, and Greenock have made arrangements for the instruction of these children, and other large towns might, at a cost that would not be serious in proportion to their size, provide teaching for the deaf in at least one school within their limits.

